# Introduction to special issue on new directions in physical rehabilitation of musculoskeletal pain conditions

**DOI:** 10.1097/PR9.0000000000000852

**Published:** 2020-09-23

**Authors:** Michele Sterling

**Affiliations:** RECOVER Injury Research Centre and NHMRC CRE in Road Traffic Injury Recovery, The University of Queensland, Brisbane, Australia

It is unequivocal that common musculoskeletal pain conditions such as back, neck, and joint pain are the leading causes of worldwide disability burden.^2^ Physical rehabilitation has always played a valuable role in the treatment of people with musculoskeletal pain. Yet, physical rehabilitation approaches, for example, exercise, have often played second fiddle to pharmacological and psychological treatments. When I attended my first IASP conference in Vienna over 20 years ago, I was disappointed that the sessions on physical rehabilitation were limited in number, attended only by physical therapy devotees, and did not feature centre stage as plenary sessions. The presented approaches seemed rather narrow, mainly involving nonspecific walking-type programs, with little consideration of the potentially stronger role that physical rehabilitation providers could play in the care of people in pain.

How things have changed. Arguably, with the emergence of the “Exercise is Medicine” movement, and the opioid overuse problem in many countries, there has been greater focus on the role of nonpharmacological approaches including exercise in the management and prevention of chronic pain. The importance of exercise for chronic conditions such as dementia, diabetes, and cardiovascular disease amongst others is clearly recognised.^1,12^ Basic scientists began investigating the effects of exercise for pain through animal studies.^8^ Clinical trials of physical rehabilitation and exercise-based approaches for musculoskeletal pain were completed with gusto, but mostly only modest effects on pain and disability were found.^13,15^ New approaches were needed and some of these are featured in this special issue.

This special issue brings together a collection of 10 articles from leaders in the field that highlight current innovations in physical rehabilitation pain research and practice including: mechanisms underpinning the effects of exercise for pain in both humans and animals, integrating psychological approaches and pain science education with more traditional exercise approaches and how these combined treatments may exert their effects, risk-stratified models of care, the role of physical therapy for peripheral neuropathies, specific challenges in indigenous populations, and perspectives of the role of physical therapy in view of the opioid crisis in some countries.

Exercise is often considered from the frame of increasing muscle strength or improving aerobic capacity. However, exercise also induces analgesic effects (exercise induced hyperalgesia [EIH])^17^ and harnessing these effects could be beneficial for people with pain. The articles by Vaegter and Jones^21^ and Lesnack and Sluka^10^ comprehensively review the mechanisms of EIH in both clinical populations and animals, respectively. The review by Vaegter and Jones^21^ demonstrates that although EIH responses are robust in pain-free individuals, they are more inconsistent in people with musculoskeletal pain, with the opposite effect of hyperalgesia with exercise often occurring. Nevertheless, EIH can be induced in patients with pain by exercising nonpainful body parts and that expectations, preferences, and beliefs can influence hypoalgesic responses and these should be assessed in determining a specific exercise intervention for individual patients. Of course, a more in-depth understanding of the effects of exercise can be gained from animal studies, where Lesnack and Sluka^10^ eloquently describe the peripheral and central mechanisms of EIH. Interestingly, they note a U-shaped curve to exercise intensity where exercise of too low or too high an intensity may have detrimental effects, suggesting that there is a sweet spot for exercise prescription that may vary between individuals.

In recent times, physical rehabilitation has embraced the biopsychosocial model and explored the addition of treatments to those that traditionally target the physical aspects of someone's pain. One of these approaches involves the including of psychological treatments delivered by nonpsychologists and termed “psychologically informed physical therapy.” Three articles in this special edition address this approach. In a narrative review, Coronado et al.^16^ explore the effects of this approach and which components contribute to make one intervention more effective than another. The authors conclude that although there is preliminary promising evidence for psychologically informed physical therapy, intensive training is necessary and implementation is problematic within current physical therapy education models. Elphinston et al.^3^ investigated mechanisms underlying how these hybrid physical therapy approaches may exert their effects. Using mediational analyses, they found that improvements in stress, depression, posttraumatic stress, and pain-related coping were causal mechanisms of effect in a physiotherapist-delivered integrated stress inoculation and exercise-integrated intervention. These results indicate that nonpsychologists can positively impact psychological symptoms with these improvements also resulting in improvements in pain and disability. Finally, Soderlund et al.^18^ undertook a scoping review of the implementation of behavioural approaches by physical therapists outlining future directions for the uptake of this approach. Another approach is the addition of Pain Neuroscience Education to usual physical therapy, which has moderate evidence of effect for patients with low back pain.^22^ In this special issue, Stanton et al.^19^ demonstrate the feasibility of combining Pain Neuroscience Education with a walking program for people with knee arthritis. We are looking forward to results of the definitive trial currently underway.

Risk-stratified models of care have increased in popularity in the past decade. This model stratifies patients with acute musculoskeletal pain for their risk of developing chronic pain and disability using validated tools. Promising effects of a risk-stratified approach have been found for low back pain, whiplash, and workers with soft tissue injury.^6,14,20^ A stepped care model is a different approach where treatment decisions are guided by response to treatment, and more comprehensive treatment is provided to people who do not respond to more straightforward care. Kongsted et al.^9^ examined the similarities and differences of these care models. They argue that the approaches are similar and that future imperatives would be to reduce the complexity of implementation by developing more standardised models that would work across conditions. This would certainly have advantages in clinical practice.

This special issue takes a broad approach to physical therapy directions. The final 3 articles cover disparate but equally important topics. Lin et al.^11^ discuss the difficulties that indigenous people have in accessing rehabilitation and how these may be overcome. George and Goode venture into the controversial opioid overuse issue in many Western countries and describe how physical therapy may be used to increase exposure to nonpharmacological treatments for people with musculoskeletal pain.^5^ Finally, Jesson et al. review the current literature on the efficacy and safety of physical therapy for peripheral neuropathies. Although physical approaches to neuropathies first emerged over 30 years ago,^4^ they are now becoming more mainstream and it is assuring to see the evidence for effectiveness increasing, although there is still much work to be done.^7^

In summary, this special issue provides only part of the picture of the exciting directions physical rehabilitation is taking to improve the treatment for people with pain. However, there is still a long way to go and much work to be done. Physical rehabilitation research and practice needs to continue to push the boundaries, challenge historical dogma, and work together to address the ever growing world burden of musculoskeletal pain.

## Disclosures

The author has no conflicts of interest to declare.
